# Machine Learning Electron Density Prediction Using Weighted Smooth Overlap of Atomic Positions

**DOI:** 10.3390/nano13121853

**Published:** 2023-06-13

**Authors:** Siddarth K. Achar, Leonardo Bernasconi, J. Karl Johnson

**Affiliations:** 1 Computational Modeling & Simulation Program, University of Pittsburgh, Pittsburgh, PA 15260, USA; ska31@pitt.edu; 2 Department of Chemical & Petroleum Engineering, University of Pittsburgh, Pittsburgh, PA 15261, USA; 3 Center for Research Computing and Department of Chemistry, University of Pittsburgh, Pittsburgh, PA 15260, USA; leb140@pitt.edu

**Keywords:** machine learning, electron density, quantum chemistry, charge transfer

## Abstract

Having access to accurate electron densities in chemical systems, especially for dynamical systems involving chemical reactions, ion transport, and other charge transfer processes, is crucial for numerous applications in materials chemistry. Traditional methods for computationally predicting electron density data for such systems include quantum mechanical (QM) techniques, such as density functional theory. However, poor scaling of these QM methods restricts their use to relatively small system sizes and short dynamic time scales. To overcome this limitation, we have developed a deep neural network machine learning formalism, which we call deep charge density prediction (DeepCDP), for predicting charge densities by only using atomic positions for molecules and condensed phase (periodic) systems. Our method uses the weighted smooth overlap of atomic positions to fingerprint environments on a grid-point basis and map it to electron density data generated from QM simulations. We trained models for bulk systems of copper, LiF, and silicon; for a molecular system, water; and for two-dimensional charged and uncharged systems, hydroxyl-functionalized graphane, with and without an added proton. We showed that DeepCDP achieves prediction $R^{2}$ values greater than 0.99 and mean squared error values on the order of $10^{-5}$$e^{2}$ Å${}^{-6}$ for most systems. DeepCDP scales linearly with system size, is highly parallelizable, and is capable of accurately predicting the excess charge in protonated hydroxyl-functionalized graphane. We demonstrate how DeepCDP can be used to accurately track the location of charges (protons) by computing electron densities at a few selected grid points in the materials, thus significantly reducing the computational cost. We also show that our models can be transferable, allowing prediction of electron densities for systems on which it has not been trained but that contain a subset of atomic species on which it has been trained. Our approach can be used to develop models that span different chemical systems and train them for the study of large-scale charge transport and chemical reactions.

## 1. Introduction

Electron density is a fundamental concept in quantum mechanics that describes the distribution of electrons in a molecule. The electron density is calculated from the solution of the Schrödinger equation, which provides a measure of the probability of finding an electron at a specific location in space. Electron density is important for the calculation of properties, such as total energy, the dipole moment, and atomic charges. The density and density differences provide insight into charge transfer, chemical reactions, types of chemical bonding, etc. Charge densities can be measured experimentally through X-ray diffraction [1], allowing for a comparison of calculated and experimental quantum mechanical information.

There are several methods that can be used for calculating electron densities. These include (Kohn–Sham) density functional theory (DFT) and the Hartree–Fock and post-Hartree–Fock methods. The cost of these calculations typically scales within the range $O(N^{3})$-$O(N^{7})$ where *N* is the number of electrons in the system, rendering these methods prohibitive for very many atoms. In addition, dynamical simulations based on molecular dynamics (MD) can only be carried for relatively short simulation times (typically no more than $10^{2}$–$10^{3}$ ps). These computational limitations make it desirable to develop alternative methods that can accurately predict molecular properties without resorting to quantum mechanical calculations.

There has been a recent surge in the application of machine learning (ML)-based algorithms applied to problems in computational chemistry and material science. A popular framework is to train regression models that behave like atomic forcefields [2,3,4,5,6,7,8,9,10,11]. These ML-based potentials make it possible to predict molecular energies using only atomic coordinates as input. Training data are commonly generated from DFT or higher-level quantum chemical calculations. Most of these methods employ featurization techniques of generating atomic descriptors that are mapped to physical properties, which are the total energy and atomic forces in most ML-based atomic potentials. These methods allow for linear-scaling MD simulations of systems with near-quantum mechanical accuracy. A similar approach can be used to predict more fundamental properties of a system, such as the electron density. Several ML techniques have been developed to map atomic coordinates onto electron densities [12,13,14,15,16,17,18,19,20]. Bogojeski et al. [12] were among the first to use an atom-centered basis set representation to machine learn 3-D electron densities. Similar atom-centered approaches were used by Fabrizio et al. [13] to study non-covalent systems and by Grisafi [14] to study hydrocarbons. Rackers et al. [16] used Euclidean neural networks with atom-centered Gaussian basis functions to train models for bulk water. Gong et al. [18] used a grid-point-based approach with crystal graph convolution neural networks to train electron densities. Similarly, Chandrasekaran et al. [15] used a grid-point-based approach with Gaussian-based fingerprints to build models for electron density and density of states (DOS) for periodic systems. To the best of our knowledge, these approaches have only addressed charge neutral systems. It remains an open question whether modeling condensed systems with positive or negative charges can be achieved with comparable levels of accuracy and efficiency. Another important aspect of electron density prediction methods that has not been addressed so far is the ability of a given model to account for only specific electron density regions, avoiding a global mapping of atomic positions to electron densities. In addition to making predictions faster and more efficient, this feature can be used to streamline the analysis of electron density-dependent physical properties, particularly during molecular dynamics simulations on large systems.

In this work, we developed a deep learning approach for charge density prediction, which we call DeepCDP. We demonstrate the use of the smooth overlap of atomic positions (SOAP) descriptors to map atomic coordinates to electron densities. SOAP descriptors constructed at atomic centers are commonly used to train ML potentials, such as the Gaussian approximation potential (GAP) [2]. We instead generated such fingerprints at spatial grid points of a system and trained a neural network (NN) to predict densities at those points. The grid-point-based approach is known to use significantly fewer training images as compared to the atom-centered basis representation approach [15]. We show the advantage of using a weighting function in SOAP to generate more sensitive fingerprints. The local nature of DeepCDP makes it possible to model large periodic systems. Our method can also model charged systems due to the addition of a special constraint that maintain the number of electrons in the system. This is of particular importance because it allows the use of our DeepCDP models in conjunction with ML potentials that we have generated for charged systems, such as hydroxyl-functionalized graphane (graphanol) [21,22]. This allows us to have a framework that can compute dynamics, geometries, energetics, and electron densities with near-DFT accuracy at a very small fraction of the computational cost required for DFT calculations.

## 2. Methods

### 2.1. Fingerprinting

Input data for training an NN was in the form of atomic descriptors that were projected as a 1-D vector of arbitrary features. We used the SOAP formalism to construct our descriptors, as implemented by De et al. [23]. SOAP encodes each region of the atomic geometry by utilizing local expansions of empirical functions, such as Gaussian-smeared atomic densities, spherical harmonics, and radial basis functions. As with most atomic descriptors, the SOAP formalism represents the atomic neighborhood of a point in space (*r*) inside a cutoff radius $r_{\mathrm{cut}}$. The partial power spectrum p encodes information on the relative arrangement of pairs of species ($Z_{1}$ and $Z_{2}$), which is written as

(1) $$p_{nn^{\prime }l}^{Z_{1}Z_{2}}=\pi \sqrt{\frac{8}{2l+1}}\sum_{m}c_{nlm}^{Z_{1}}c_{n^{\prime }lm}^{Z_{2}},$$

where $c_{nlm}^{Z}$ are coefficients that are computed as inner products of the above-mentioned empirical functions. Indices for different radial basis functions are labeled by *n* and $n^{\prime }$, up to $n_{\max}$. The angular degree of the spherical harmonics is indicated as *l*, which can range to $l_{\max}$. Parameters such as $n_{\max}$ and $l_{\max}$ are chosen by the user. High values of $n_{\max}$ and $l_{\max}$ will result in larger p, which allows for more detailed fingerprinting. However, larger p vectors will increase the time needed to train ML models. The magnetic quantum number is labeled as *m*. We generate the vectors p using a grid-point basis, rather than using an atom-centered basis. The coefficients are given by

(2) $$c_{nlm}^{Z}=\underset{R^{3}}{\;\int \int \int \;}dVg_{n}\left(r\right)Y_{l}^{m}\left(\theta ,\phi \right)\rho^{Z}\left(r\right),$$

where $g_{n}\left(r\right)$ are radial basis functions. We chose Gaussian-type orbitals to define $g_{n}\left(r\right)$. $Y_{lm}\left(\theta ,\phi \right)$ are the spherical harmonics. We used a weighting function w(r), which should ideally be included in the integrand. However, this complicates the calculation of the integral, and we therefore included w(r) in the function that defines the Gaussian-smeared atomic densities

(3) $$\rho^{Z}\left(r\right)=\sum_{i}^{\left|Z_{i}\right|}w\left(\left|R_{i}\right|\right)e^{-1/2\sigma^{2}{\left|r-R_{i}\right|}^{2}},$$

where σ is the standard deviation of the Gaussian densities. We chose the polynomial form of w(r), [24] which is defined as

(4) $$w\left(r\right)=\left\{\begin{array}{c} c{\left[1+2{\left(\frac{r}{r_{0}}\right)}^{3}-3{\left(\frac{r}{r_{0}}\right)}^{2}\right]}^{m},\;\mathrm{for}\;r\leq r_{0}\\ 0,\;\mathrm{for}\;r>r_{0} \end{array}.\right.$$

Note that $r_{0}$ and $r_{\mathrm{cut}}$ can be different. The optimal set of parameters that define w(r) (*c*, *m*, and $r_{0}$) can vary from one kind of system to another. Including the weighting function in the SOAP formalism helps with radially distributing the scale of atomic densities, thus giving more weight to atoms that are closer to any point *r* [25]. Without such a weighting function, we observe instances where the p vectors for different points in space are nearly indistinguishable, as will be discussed in the Results section. We used the DScribe Python package [26] to construct our p vectors.

### 2.2. NN Training

**Figure 1:**
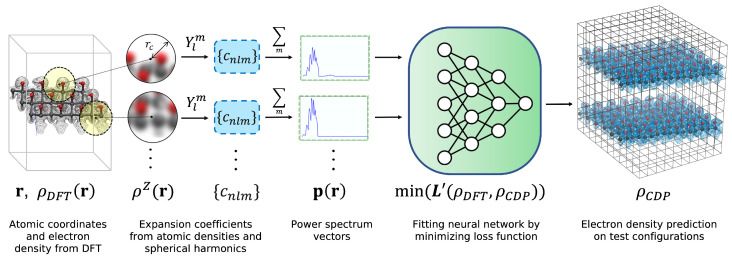
Overall schematic of the process of building a DeepCDP model. The first step involves generating DFT training data that are composed of atomic coordinates for a system and the corresponding electron density $\rho_{DFT}\left(r\right)$ at each grid point r. Other quantum mechanics methods can also be used to generate training data and are not limited to DFT. The next step is to generate grid-based fingerprints with weighted SOAP by just using the atomic coordinates as input. Gaussian-smeared atomic densities $\rho^{Z}\left(r\right)$ and spherical harmonics $Y_{l}^{m}$ are used to generate expansion coefficients $c_{nlm}^{Z}$. These coefficients are used to compute the partial power spectrum **p** for each point r. These vectors are then used to train an NN by minimizing a loss function $L^{\prime }$. The loss function $L^{\prime }$ is defined as the error in the NN’s prediction ($\rho_{CDP}\left(r\right)$) compared to $\rho_{DFT}\left(r\right)$. The converged NN is tested on unseen configurations and then used to generate electron density data ($\rho_{CDP}\left(r\right)$) for new systems.

In this work, we obtained grid-based electron densities $\rho_{DFT}\left(r\right)$ from DFT. However, we note that generation of training data does not require the use of DFT. One could use high-accuracy wavefunction methods or even experimental data to train and evaluate DeepCDP. The process of generating training data, constructing fingerprints using weighted SOAP (p), training and testing a DeepCDP model is illustrated in Figure 1. We constructed our NNs via the Pytorch [27] package. We also used the Multi-layer Perceptron (MLP) regressor implementation in scikit-learn [28] to build a few simple models for testing purposes. The input layer of the NN takes into account the p vector for a given point in space *r*. We built a cylindrical NN that contained 3 hidden layers having 300 neurons each. This architecture was used throughout for all examples. We used rectified linear units (ReLu) [29] non-linear activation between layers of each NN. We also used batch normalization [30] and induced dropout of some neurons to increase robustness of the models. Each p for a given *r* was mapped to its corresponding scalar “true” electron density $\rho_{DFT}\left(r\right)$. The loss function (*L*) was estimated using either the mean absolute errors (MAEs) or the mean squared errors (MSEs), based on the system. We started each NN optimization with MSE because it converged quickly toward the answer, but then we switched to MAE to improve convergence in the vicinity of the solutions. MSE is known to penalize large errors and outliers and so we found prediction accuracies to quickly improve and then plateau. Switching to MAE then allowed us to fine-tune the predictions as it linearly weights all errors and outliers at that point were few. We used the stochastic optimization method (Adam) [31] to minimize the loss function. The learning rate for the loss function was dynamically reduced during training when the value of loss plateaued. We used a weight decay of $1\times 10^{-4}$ for regularization. We found that just using MAE or MSE as the loss function did not correctly predict the total number of valence electrons. We therefore modified the loss function *L* by adding a constraint for the total number of valence electrons in the system:

(5) $$L^{\prime }\left(\rho_{DFT},\rho_{CDP}\right)=L\left(\rho_{DFT},\rho_{CDP}\right)+\alpha \left|\sum \rho_{DFT}\left(r_{i}\right)-\sum \rho_{CDP}\left(r_{i}\right)\right|dV$$

where $L^{\prime }$ is the modified loss function, *L* is either MAE or MSE, a prefactor α was set to 1.0 for MAE and 0.1 for MSE, $\rho_{DFT}$ are the DFT electron densities, $\rho_{CDP}$ are the densities predicted by DeepCDP, and dV is the differential volume.

### 2.3. Data Generation with DFT

We generated our training data by randomly sampling configurations from density functional theory molecular dynamics (DFT-MD) simulations. We performed simulations for five different systems: a bulk metal (Cu), a semiconductor (crystalline Si), a wide band-gap insulator (LiF), a molecular fluid (water), and a 2-D system (graphanol, or hydroxyl-functionalized graphane [21,22,32,33]). These calculations were performed using the Quickstep [34] module in the CP2K package [35]. DFT simulations to generate electron density data for water were performed using the BLYP [36] generalized gradient approximation (GGA) exchange-correlation functional with D3 dispersion corrections [37]. The BLYP functional is traditionally used to model liquid water and is also shown to give good results when compared to experiments [38]. We used the Perdew–Burke–Ernzerhof (PBE) [39] GGA functional for all other systems. The PBE functional is commonly used in solid-state systems. The hybrid Gaussian and plane-wave method [40] was used. DZVP basis sets [41] with GTH pseudopotentials [42] were employed for water. All other systems used the DZVP-MOLOPT-SR basis sets with the GTH pseudopotentials. The focus of our work is to show that DeepCDP predicts electron densities with the same accuracy as DFT and so we are not primarily concerned with the accuracy of the DFT calculations themselves. The choice of functionals in our work reflects common choices from the literature for specific systems. We used a Monkhorst–Pack k-point grid size of 2×2×2 for the Brillouin zone sampling in bulk Cu and Si. We used only the Γ-point for all other systems.

DFT-MD simulations were performed within the NVT (canonical) ensemble using a GLE thermostat [43]. We used MD simulations of 25 ps to generate data. Data generation for Cu, Si, LiF, and graphanol was performed at T=1000 K. Data generation for water was performed at T=298 K. The size of the training data was dependent on the total number of grid points that were selected. Smaller systems such as bulk Cu (containing 2 atoms in its primitive cell) had just over 5000 grid points per training image. We used just 10 DFT images to train models for Cu, which resulted in a total of over 50,000 training data points. Larger systems such as graphanol with 61 atoms in the cell had 324,000 grid points per image. We used just 6 images in our training data set, which led to a total of over 1.9 million data points. All our models were trained on an Apple M1 GPU. Total training time depended on the system size that was used and the p vector. Simpler systems such as bulk Cu with fewer p vector features per data point (180) took less than 8 s per training epoch. Larger systems such as graphanol with larger p vector features per data point (390) took about 110 s per training epoch. For comparison, we tested these systems on an 8-core CPU and found training to take 15 s per epoch for the bulk Cu example and 170 s per epoch for the graphanol example.

## 3. Results and Discussion

### 3.1. Bulk Cu

**Figure 2:**
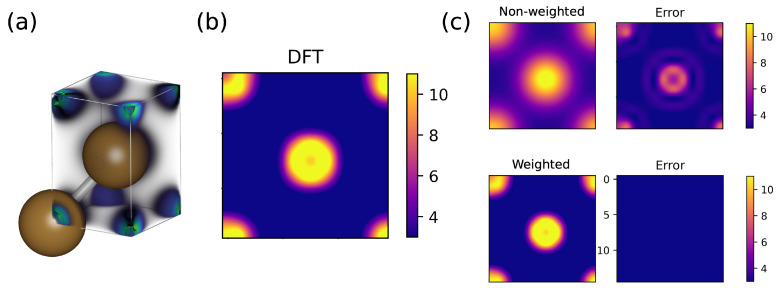
Comparing the importance of weighting function in SOAP. (**a**) Snapshot of the bulk Cu system with a 3-D plot of the DFT electron density. (**b**) Contour plot of DFT electron density integrated along the *z* axis. (**c**) DeepCDP-predicted electron densities and errors when trained using non-weighted SOAP (top row) and weighted SOAP (bottom). Color bar units are in *e* Å${}^{-3}$.

We first tested the importance of the polynomial weighting function, w(r), Equation (4), that is added to the definition of SOAP. This testing was performed by comparing two NNs for Cu that were trained with and without the weighting function. A periodic face-centered cubic crystal structure of Cu (Figure 2a) was used to generate our models.

We performed preliminary tests to check for the sensitivity of SOAP, generated with and without weighting, by sampling points along a line connecting two Cu atoms. We observed that non-weighted SOAP appeared less sensitive to where these vectors were generated compared to weighted SOAP. Features that are less sensitive to the location of the point relative to the atom can make it harder for the NN to converge. We estimated the sensitivity of these SOAP vectors relative to each other by computing the quantity

(6) $$S=\frac{\min_{i\neq j}∥p_{i}-p_{j}∥}{\max_{i}∥p_{i}∥},$$

where $p_{i}$ and $p_{j}$ are two different SOAP vectors in the given set of SOAP vectors, and ∥·∥ denotes the $L_{2}$ norm (i.e., the Euclidean distance). The *S* value is the ratio between the smallest separation between any two vectors in our set of $p_{j}$ vectors (numerator) and the largest magnitude among all the vectors in that set (denominator). This ratio is a measure of how much the vectors in our set are influenced by changes in their positions relative to their sizes. So, a high *S* value indicates that even a small change in the vector positions can have a significant impact on the overall vector magnitude. Using non-weighted SOAP fingerprints resulted in an *S* score of $7.3\times 10^{-4}$, while using weighted SOAP fingerprints yielded a score of 0.13. A score close to zero indicates that fingerprints generated at different points in space are identical to each other. These results show that a model trained on non-weighted SOAP fingerprints will converge less easily than a model trained on weighted SOAP. We then conducted a practical test by comparing two NN models that were trained with and without the weighting function. The architecture, data set, and training protocol of the two NN models were exactly the same, as discussed in the Methods section. We used a data set containing electron density data from 10 DFT-MD images of bulk Cu (with 2 atoms per cell, as shown in Figure 2a). The electron density prediction accuracy of these models was assessed using test configurations that were not part of the training set. The contour plot of $\rho_{DFT}$ integrated along the *z* axis for a given snapshot is shown in Figure 2b. The predictions from the model trained with weighted SOAP and the corresponding absolute error relative to DFT are shown in the top row of Figure 2c. The results from the weighted SOAP model are shown in the bottom row of Figure 2c. It is clear that using the weighting function has an advantage for mapping out the 3-D electron density function. We compared the $R^{2}$ and MSE values for the predictions of these two models. These results are reported in Table 1. The weighted SOAP model gives an $R^{2}$ value of 0.991 for the predicted densities. We also see more than an order of magnitude reduction in the MSE with the weighted SOAP model. These results demonstrate that weighting of the SOAP function is critical to achieving high accuracy.

### 3.2. Bulk Si and LiF

**Figure 3:**
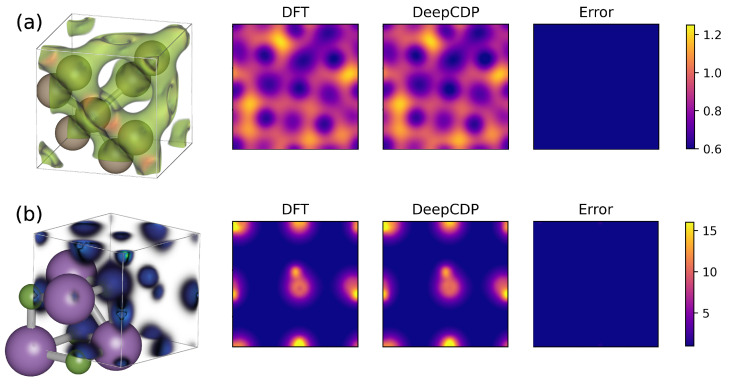
Comparing electron density predictions for Si and LiF. (**a**) (Left to right): Snapshot of the bulk Si system (light brown atoms) with a 3-D plot of the DFT electron density (green). Contour plot of the DFT electron density integrated along the *z* axis. Contour plot of the DeepCDP electron density integrated along the *z* axis. Contour plot of prediction error of DeepCDP integrated along the *z* axis. (**b**) (Left to right): Snapshot of bulk LiF system (Li atoms in green, F atoms in purple) with a 3-D plot of the DFT electron density. Contour plot of DFT electron density integrated along the *z* axis. Contour plot of the DeepCDP electron density integrated along the *z* axis. Contour plot of prediction error of DeepCDP integrated along the *z* axis. Color bar units are *e* Å${}^{-3}$.

We tested our DeepCDP formalism on two other periodic bulk systems, Si and LiF. We chose these systems because of qualitative differences in their electron density distribution compared to Cu. Bulk Si is a semiconductor with covalent Si-Si bonds, which results in electron density accumulation along the bonds. LiF is a wide-gap insulator with ionic bonds, which results in electrons being more localized in the vicinity of the nuclei. We generated training data for both these systems and trained models for LiF and Si. We used the same NN architecture as the one used for Cu. The electron densities from DFT integrated along the *z* axis and the corresponding DeepCDP predictions and absolute errors for the two systems are plotted in Figure 3. We obtained an $R^{2}$ value of 0.996 and 0.998 on the test data for Si and LiF, respectively. Their corresponding MSEs are $3.3\times 10^{-6}$ and $2.2\times 10^{-5}$ $e^{2}$ Å${}^{-6}$, respectively. These values of the $R^{2}$ and MSE indicate that generating descriptors using weighted SOAP can accurately describe systems having very different electron density profiles.

### 3.3. Scaling with System Size

**Figure 4:**
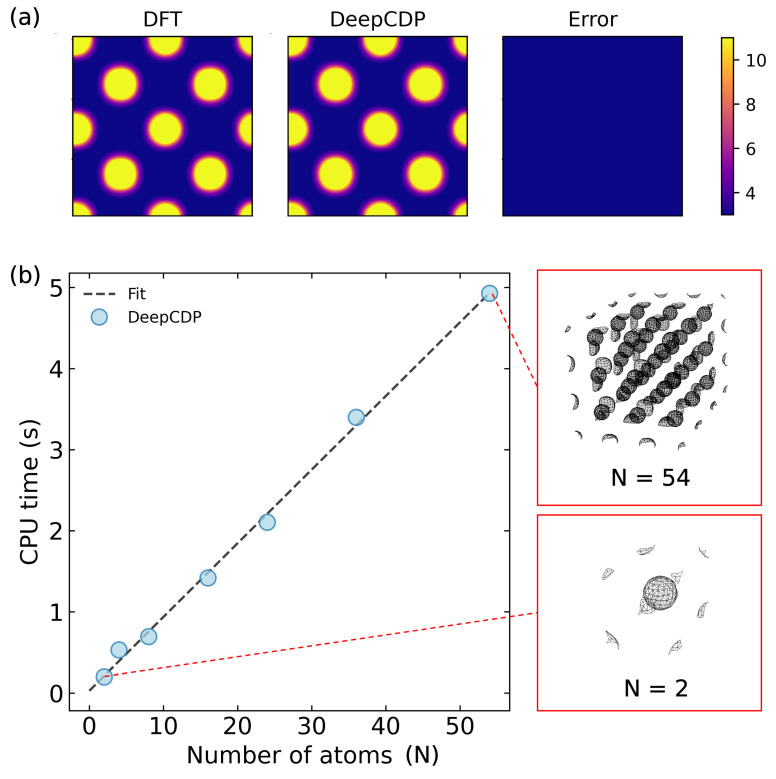
(**a**) (Left to right): Contour plot of DFT electron density integrated along the *z* axis for a 2×2×2 supercell of bulk Cu. Contour plot of DeepCDP electron density predictions for the same system. Contour plot of prediction error between DFT and DeepCDP integrated along the *z* axis. Color bar units are *e* Å${}^{-3}$. (**b**) (Left): DeepCDP electron density compute times as a function of the number of Cu atoms, *N* (circles). Linear fit (dashed line) of the data points. (Right): Images of DeepCDP electron density predictions for N = 2 (bottom) and N = 54 (top).

We used the model built for Cu to test it on larger system sizes. The test data containing 10 images of a 2×2×2 Cu supercell with 16 atoms was generated with DFT. The integrated density plots are shown in Figure 4a. We observed an $R^{2}$ value of 0.997 and an MSE of $4.4\times 10^{-4}$ $e^{2}$ Å${}^{-6}$. These results demonstrate that NNs trained on small system sizes can be used to predict densities for much larger system sizes, which have atomic configurations not observed in the small systems, without a loss of accuracy. This is made possible by the local nature of the SOAP fingerprints.

In addition to accuracy, we tested the computational scaling with increasing system size. We observed linear scaling with the number of atoms in the system. We used the Cu model to perform these tests. The scaling relation is shown in Figure 4b. We collected the compute times for systems containing from 2 to 54 atoms. The number of grid points increases linearly with the number of atoms in the system, which explains the linear scaling for the compute time.

### 3.4. Water

**Figure 5:**
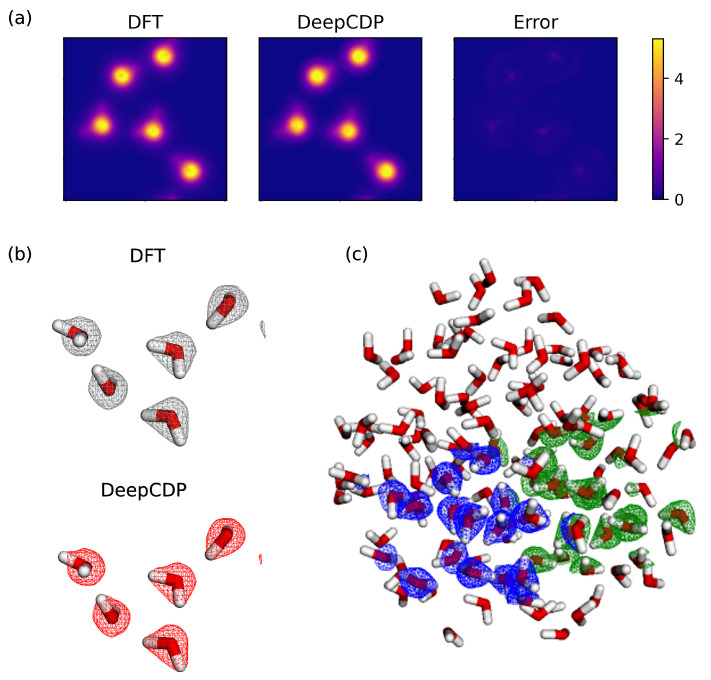
(**a**) (Left to right): Contour plot of DFT electron density integrated along the *z* axis for a system containing five water molecules. Contour plot of DeepCDP electron density prediction for the same system integrated along the *z* axis. Contour plot of prediction error between DFT and DeepCDP integrated along the *z* axis. Color bar units are in *e* Å${}^{-3}$. (**b**) (Top) 3-D electron density isosurface plots from DFT (grey) and (bottom) DeepCDP (red). An isosurface value of 0.08 au was used. Red indicates oxygen atoms and white indicates hydrogen atoms. (**c**) DeepCDP electron density prediction isosurfaces of small sections (blue and green) of a large cell containing 139 water molecules. An isosurface value of 0.08 au was used. Each section contains 16 water molecules.

The systems considered so far were simple bulk periodic structures that are highly ordered. Predicting electron densities for dynamically amorphous systems, such as liquids, can be more challenging due to the absence of symmetry. We generated structures and electron densities from DFT-MD simulations of a very small system consisting of 5 water molecules in a periodic simulation cell at T=298 K and a fluid density of about 300 kg/m^3^. Note that this density is within the two-phase region of bulk liquid water, but the system is a homogeneous fluid because the simulation time is too short to allow for phase segregation. We used 10 snapshots from this simulation to build a DeepCDP model for fluid water. We trained our NN by switching between the MAE and MSE loss functions. We found that the model was able to fine-tune its predictions because of the use of these two loss functions together. Our tests are shown in Figure 5a. We obtained an $R^{2}$ value of 0.996 and an MSE value of $2\times 10^{-5}$$e^{2}$ Å${}^{-6}$ for the test data. A comparison of the DFT and DeepCDP density isosurfaces is shown in Figure 5b. To demonstrate the local nature of the densities predicted from DeepCDP, we computed electron densities for 2 regions of the water molecules taken from a snapshot of a simulation containing 139 water molecules. This large structure was obtained by selecting 139 molecules of water from a classical simulation containing 266,667 water molecules [44]. Each region contains 16 water molecules. The electron densities were generated from two independent DeepCDP calculations. Figure 5c shows the predictions of the two connected regions, with the blue mesh giving the density isosurface from one calculation and the green mesh from the other calculation. This comparison shows that our approach can be used to estimate electron densities for specific regions of a large system in parallel and independently.

### 3.5. Graphanol

**Figure 6:**
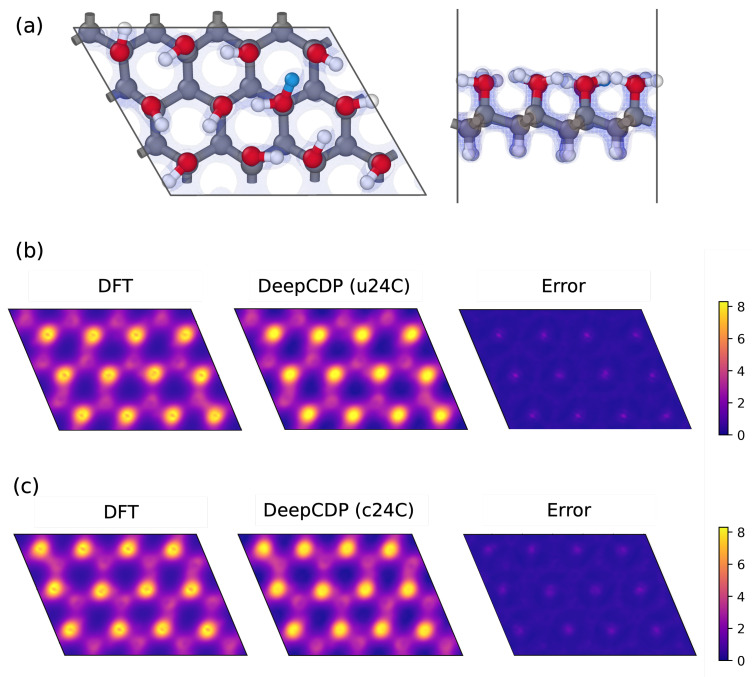
(**a**) Top (left) and side (right) view of charged graphanol (c24C). Grey indicates carbon atoms, red indicates oxygen atoms, and white indicates hydrogen atoms. Proton is highlighted as blue hydrogen atom. (**b**) Predictions from model trained with u24C data. (Left to right): Contour plot of DFT electron density integrated along the *z* axis of a c24C snapshot. Contour plot of DeepCDP electron density prediction of same configuration integrated along the *z* axis. Contour plot of prediction error between DFT and DeepCDP (u24C) integrated along the *z* axis. (**c**) Predictions from model trained with c24C data. (Left to right): Contour plot of DFT electron density integrated along the *z* axis of a c24C snapshot. Contour plot of DeepCDP electron density prediction of same configuration integrated along the *z* axis. Contour plot of prediction error between DFT and DeepCDP (c24C) integrated along the *z* axis. Color bar units are in *e* Å${}^{-3}$.

It has been shown that graphanol is a promising material for use in proton exchange membrane fuel cells [21,22,32,33]. As such, graphanol may either be neutral or positively charged due to the addition of protons. We therefore used graphanol to assess the ability of DeepCDP to predict electron densities for charged systems and to track the location of the excess charge. We generated DFT data for 2 graphanol systems: u24C, which is uncharged graphanol with 24 carbon atoms, and c24C, which is the same as u24C but has a net positive charge due to the addition of 1 proton. We trained two DeepCDP models, one for each of these systems. Figure 6a contains the top and side views of c24C, where the added proton is depicted as a blue atom. The results from our predictions are reported in Table 2. Both of these models were capable of achieving high $R^{2}$ values and low MSE values on the test data. Figure 6b,c show the *z*-axis integrated electron densities of the c24C system predicted using the two models. We used the total number of predicted valence electrons as a metric to test for the model’s ability to account for charges. We found that the model trained using only u24C data overestimated the total number of valence electrons in c24C. This is because the model treats an added proton as a hydrogen atom. However, the model trained using c24C data did not overestimate the number of valence electrons in c24C and correctly predicted that the total number of valence electrons in u24C and c24C are the same.

**Figure 7:**
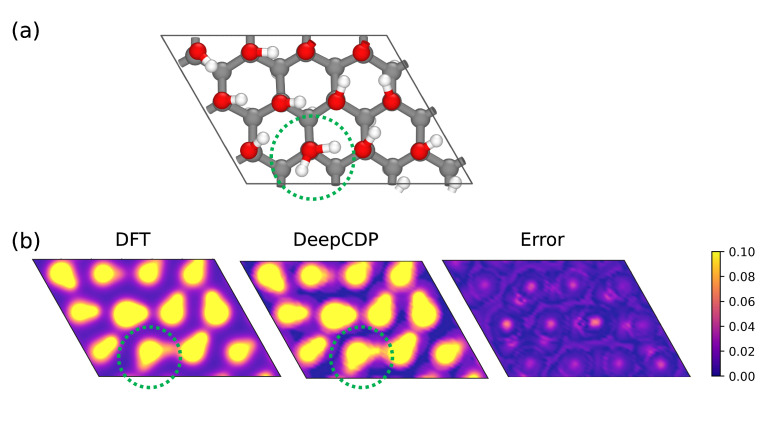
(**a**) Graphanol c24C structure. The green dotted circle indicates the location of the proton. (**b**) Contour plots of electron densities on the *x*-*y* plane showing hydrogen bonding in (**a**) from DFT (left) and the c24C DeepCDP model (middle). Errors in prediction between DFT and DeepCDP (right). Note that we used a narrow data range (0–0.1 *e* Å${}^{-3}$) to highlight differences. Color bar units are in *e* Å${}^{-3}$.

The c24C model is capable of correctly identifying hydrogen bonds for the test c24C configuration, as shown in Figure 7. The contour plots show electron densities in the *x*-*y* plane that clearly indicate the location of hydroxyl groups and the proton. We used a narrow data range of 0 to 0.1 *e* Å${}^{-3}$ to highlight the errors and differences in the predictions from DFT and DeepCDP. We observe small prediction errors in DeepCDP around the voids of the material and at the centers of most oxygen atoms. Note that the color bar has a narrower electron density range compared to previous figures.

#### 3.5.1. Charge Tracking

The ability to accurately predict electron densities for charged systems from DeepCDP can be utilized to locate the position of a positively or negatively charged moiety, such as a proton or a hydroxyl group. This information can be used to track proton dynamics. Popular methods such as the Density-Derived Electrostatic and Chemical (DDEC) approach [45,46] make use of the charge density data for the entire system to calculate net atomic charges on each atom in the system. These can be used to model charges in classical simulations. Our approach is, in principle, capable of tracking charge migration without explicitly mapping electron densities onto classical charges for all atoms.

**Figure 8:**
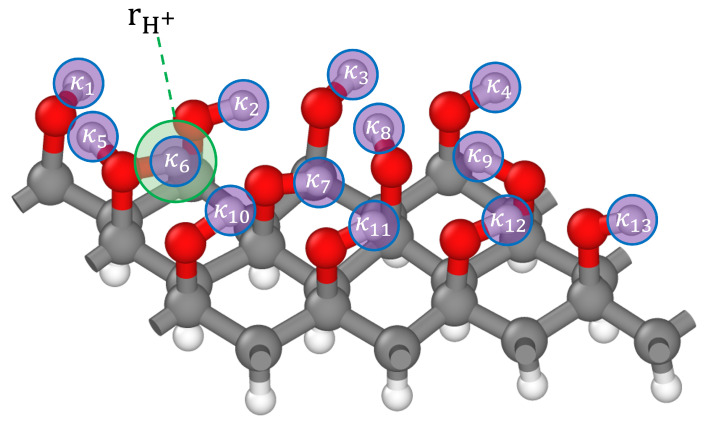
Illustration of using DeepCDP to locate the position of a proton ($r_{H^{+}}$) in charged graphanol (c24C). DeepCDP is used to calculate the electron density for points around each hydrogen atom attached to an oxygen atom. Seven points are sampled for each hydrogen atom, including the center of the atom, to compute the differential charge $\kappa_{i}$ for a given hydrogen atom *i*, computed from Equation (7). This is depicted as purple circles around each hydrogen atom. The proton (light green circle) is identified as the atom having the smallest value of κ.

Here, we assess whether our model can be used to identify if one of the hydrogen atoms in graphanol carries a positive charge, rather than predicting electron density data for the entire system. The local nature of the model allows us to predict the electron density values only at points that are relevant to the estimation of the hydrogen atom charges, which substantially reduces the computational cost. This is particularly important for dynamical simulations, which involve electron density predictions for large numbers of atomic configurations. Given the case of charged graphanol (c24C), we show as proof of concept that we can locate the position of the proton using only the atomic coordinates as input to a DeepCDP model. We make the assumption that the position of the excess positive charge will always correspond to the coordinates of one of the hydrogen atoms. For this purpose, we collected the coordinates $r_{H_{i}}$ for all the hydrogen atoms. We consider subsets of points, and we consider sets of points, $s(r_{H_{i}})$, where each set contains the position of a given hydrogen atom $r_{H_{i}}$ and six points that are the closest to $r_{H_{i}}$. These 6 closest points were sampled based on differential distances (dx=0.32 Å, dy=0.28 Å, and dz=0.31 Å), which were obtained from the DFT cube files for c24C. Other values for differential distances can also be used and are not limited to the ones we mentioned above. If we have a hydrogen atom *a* at $r_{H_{a}}$ = $\left(x_{a},y_{a},z_{a}\right)$, then the seven points in set $s(r_{H_{a}})$ are $\left(x_{a}+dx,y_{a},z_{a}\right)$, $\left(x_{a}-dx,y_{a},z_{a}\right)$, $\left(x_{a},y_{a}+dy,z_{a}\right)$, $\left(x_{a},y_{a}-dy,z_{a}\right)$, $\left(x_{a},y_{a},z_{a}+dz\right)$, and $\left(x_{a},y_{a},z_{a}-dz\right)$. We then define a differential charge $\kappa (r_{H_{i}})$ by summing the numerical values of the electron density at these sampled points,

(7) $$\kappa \left(r_{H_{i}}\right)=\sum_{i\in s}\rho_{CDP}\left(i\right),$$

where $\rho_{CDP}\left(i\right)$ is the electron density predicted at point *i* by DeepCDP. We identify the location of the proton $r_{H^{+}}$ with the coordinates of the hydrogen atom having the minimum $\kappa (r_{H_{j}})$. An illustration of this procedure for a single snapshot of graphanol c24C is shown in Figure 8.

**Figure 9:**
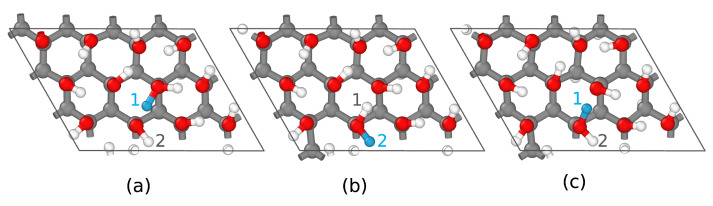
Sample trajectory of using DeepCDP to locate protons during transfer in c24C. (**a**–**c**) are sampled trajectories from a deep learning potential MD simulation. The proton is depicted as a blue atom. (**a**) DeepCDP predicts hydrogen atom 1 as the proton. (**b**) Hydrogen atom 2 is labeled as the proton after a proton hop. (**c**) Hydrogen atom 1 is labeled as the proton again as a result of changes in bond lengths.

This procedure can be repeated for series of atomic coordinates obtained from dynamical simulations. The system that we considered is c24C graphanol, as shown in Figure 6a, which contains a single added proton to one side of the material. We generated a series of 21,400 configurations from a deep learning atomistic potential classical MD simulation [21]. For each of these configurations, we obtained $r_{H^{+}}$ based on $\kappa (r_{H_{i}})$. We observed that our $r_{H^{+}}$ before the hopping corresponds exactly to the H atom that undergoes hopping. An example is provided in Figure 9a–c. Our method first indicates hydrogen atom 1 as the proton, which is accurate as it is bonded to a charged oxygen atom. We then saw that hydrogen 1 hops to the oxygen atom to which it was previously hydrogen bonded. For a brief period, we found that hydrogen atom 2 becomes the proton (Figure 9b), which then rapidly reverts to hydrogen 1 (Figure 9c) as a result of differences in the O-H bond lengths. We also saw that hydrogen 1 takes part in another proton hopping event after a period of 2.5 fs. However, there were cases where our method sometimes labeled the wrong hydrogen atom as the proton. This occurred immediately after a proton hopping event for a short duration of 0.25–1 fs (our MD simulation employed a time step of 0.25 fs). Other instances of such labeling were when minor charge fluctuations induced a temporary hop between two uncharged hydroxyl groups. For the purpose of proton tracking, these issues can potentially be filtered out by the imposition of constrains. However, the formulation of these constraints is beyond the scope of our current work.

#### 3.5.2. Model Transferability

**Figure 10:**
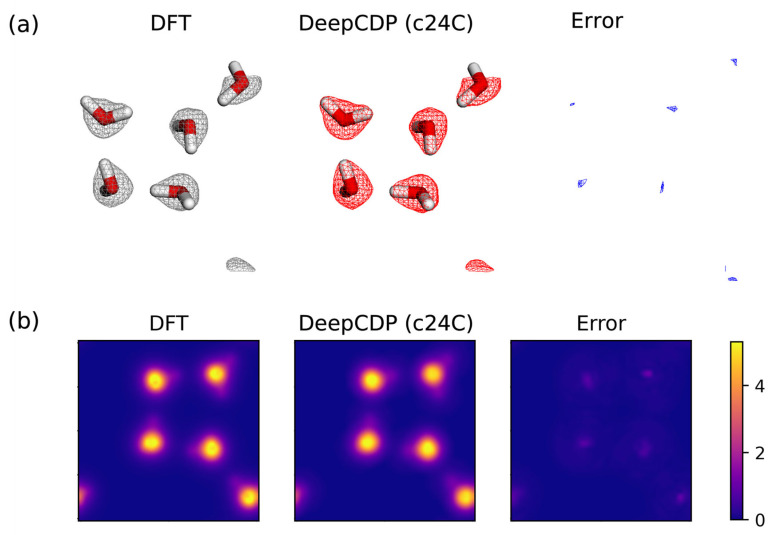
Testing model transferability by computing the density of water from a DeepCDP trained on c24C graphanol data. (**a**) (Left to right): Three-dimensional electron density isosurface plots from DFT (grey), DeepCDP (red), and the error (blue). An isosurface value of 0.08 au was used. (**b**) (Left to right): Contour plot of DFT electron density corresponding to configuration in (**a**) integrated along the *z* axis. Contour plot of DeepCDP electron density prediction for the same system integrated along the *z* axis. Contour plot of prediction error between DFT and DeepCDP integrated along the *z* axis. Color bar units are in *e* Å${}^{-3}$.

It is important to assess the transferability of DeepCDP, i.e., how well it predicts electron densities for systems on which it was not trained. For this purpose, we used our DeepCDP model for charged graphanol to predict the electron densities and the total number of electrons for a snapshot of five water molecules. Note that this model was not trained on any water data, but because graphanol contains O-H and H-O-H moieties, there is a reasonable expectation that the charge density for water might be predicted with reasonable accuracy by the DeepCDP trained on graphanol. Therefore, we used the SOAP function for c24C graphanol to predict the electron density of water. The dimensions of p from the SOAP function were unaltered for the water test case. We used test configurations from our water example as shown in Figure 5a,b. Our model for graphanol predicted electron densities of water with an $R^{2}$ value of 0.993 and an MSE value of $5\times 10^{-5}$ $e^{2}$ Å${}^{-6}$. Images containing a 3-D rendering of electron density isosurfaces from DFT and DeepCDP, error isosurfaces, and *z*-axis integrated contour plots for a test case prediction are shown in Figure 10. These results are promising as they showcase the model’s transferability to new systems. However, we found that our model yields an average of 40.8 valence electrons for the water system as compared to the DFT value of 40.2 electrons. The difference between DeepCDP and DFT may be attributed to two factors. First, to compute a reliable total number of valence electrons by integrating electron density maps requires a sufficient number of 3-D grid points. The fact that the integrated number of electrons in DFT is not exactly 40 indicates that the real-space grid used to represent the electron density, which is determined by the plane-wave kinetic energy cutoff used in the calculations, is too coarse to yield reliable electron density distributions. Second, the training of the model may need to include configurations of the target system to attain sufficient accuracy. Despite the error in the total number of valence electrons predicted, the main outcome of this exercise is to show that DeepCDP has some degree of transferability. In principle, it may be possible to develop a completely transferable DeepCDP by constructing a universal SOAP function that can account for any element of the periodic table, training the NN with data for a wide variety of chemical systems.

## 4. Conclusions

We have presented a machine learning approach that allows one to predict electron densities with near-quantum mechanical accuracy for various systems. We have validated our method by successfully predicting charge densities for a bulk metal, Cu; a covalently bound semiconductor, bulk Si; an ionic insulator, LiF; an inorganic molecular fluid, H_2_O; and an organic-like 2-D material, graphanol. We used a grid-based approach with weighted SOAP fingerprints to train and predict electron density data. We showed that our models trained on DFT data can accurately predict electron densities for a wide variety of materials, including metals, semiconductors, insulators, charged graphanol, and water. The inclusion of a constraint that maintains the total number of predicted valence electrons allowed our models to accurately account for the presence of excess charge. We utilized the power of SOAP and the ability to predict charges to showcase two interesting applications: charge tracking and transferability. The local nature of these models allows one to make density predictions for any sub-volume or set of grid points within the system. Our method can be used in conjunction with deep learning atomistic potentials to predict the dynamics, energies, forces, electron densities, and charge transport with near-DFT accuracy, orders of magnitude faster than using DFT. We showed that DeepCDP that is trained on one material can be used to predict the charge density of a different material having a subset of atoms belonging to the first material. We demonstrated transferability by using the DeepCDP model for graphanol to predict the charge density for water molecules with high accuracy. This indicates the possibility that one may be able to develop a universal DeepCDP by constructing weighted SOAP functions for a very large number of atom types.

## Figures and Tables

**Table 1:** Comparing $R^{2}$ and MSE values for electron density prediction using two models that were trained without and with weighting applied to SOAP.

Model	$R^{2}$	MSE ($e^{2}$ Å${}^{-6}$)
Non-weighted SOAP	0.619	$4.8\times 10^{-2}$
Weighted SOAP	0.991	$9.8\times 10^{-4}$

**Table 2:** Comparing $R^{2}$, MSE values of electron density prediction, and total number of predicted electrons using two models that were trained with u24C and c24C data, respectively. The units of MSE are $e^{2}$ Å${}^{-6}$.

Model	$R^{2}$ (MSE) on u24C Data	$R^{2}$ (MSE) on c24C Data	Total DFT Valence Electrons (DeepCDP Valence Electrons) in u24C	Total DFT Valence Electrons (DeepCDP Valence Electrons) in c24C
u24C	0.993 ($6.0\times 10^{-5}$)	0.989 ($6.0\times 10^{-5}$)	192.0 (192.1)	192.0 (193.2)
c24C	0.992 ($5.0\times 10^{-5}$)	0.994 ($6.0\times 10^{-5}$)	192.0 (191.8)	192.0 (192.2)

## Data Availability

Data available in a publicly accessible repository. Our publicly available codes and data sets can be found at https://github.com/siddarthachar/deepcdp.

## Abbreviations

The following abbreviations are used in this manuscript:

DeepCDP	Deep Learning for Charge Density Prediction
CDP	Charge Density Predictor
DFT	Density Functional Theory
MD	Molecular Dynamics
NN	Neural Network
SOAP	Smooth Overlap of Atomic Orbitals
MAE	Mean Absolute Error
MSE	Mean Squared Error
u24C	Uncharged Graphanol with 24 Carbon Atoms
c24C	Charged Graphanol with 24 Carbon Atoms
